# SMART LIFE, FITNESS, WELLNESS, AND THE PROMISE OF DIGITAL HEALTH TECHNOLOGIES FOR OLDER PEOPLE

**DOI:** 10.1093/geroni/igz038.084

**Published:** 2019-11-08

**Authors:** Stephen Katz

**Affiliations:** Trent University, Toronto, Ontario, Canada

## Abstract

Based on the author’s ethnographic observation and collection of product media kits, videos, and photographs from the Consumer Electronics Show 2019, this paper critiques the ways in which positive lifestyle concepts such as ‘smart life’, ‘fitness’ and ‘wellness’ are designed within products aimed at senior marketing to shape the older consumers as composites of health problems open to technological intervention. However helpful sensor clothing, home surveillance cameras, self-tracking appliances, robotic companions, or digital mobility devices may be, they are also opportunities to capitalize on shared personal data and subscription-based monitoring services. Discussion links these concepts to wider concerns about algorithimic standardization of health risks for older people, healthcare austerity programs, and social inequalities based on technical markers of successful aging and privileged life-course trajectories

